# The two faces of *Coprinus comatus*—Functional properties and potential hazards

**DOI:** 10.1002/ptr.6741

**Published:** 2020-05-27

**Authors:** Patryk Nowakowski, Sylwia K. Naliwajko, Renata Markiewicz‐Żukowska, Maria H. Borawska, Katarzyna Socha

**Affiliations:** ^1^ Department of Bromatology, Faculty of Pharmacy with the Division of Laboratory Medicine Medical University of Bialystok Bialystok Poland

**Keywords:** *Coprinus comatus*, functional properties, hazards, mushroom, shaggy mane

## Abstract

Mushrooms have been used for centuries not only as food but also in traditional medicine as a source of components with pro‐health activity. One of them is *Coprinus comatus* (O.F.Müll.) Pers. also called shaggy mane, chicken drumstick mushroom, or lawyer's wig. In Asian countries, *C*. *comatus* (CC) is approved as edible mushroom and often cultivated for consumption, whereas in many other countries, although it is widespread, it is unrecognized and not used. In this review, for the first time, we discussed about the composition related to functional properties as well as the potential risks associated with consumption of CC by reviewing scientific literature. The information has been collected in order to get to know this species thoroughly. Various studies show many of the physiological activities, such as antioxidant, anticancer, antiandrogenic, hepatoprotective, acetylcholinesterase inhibitory, antiinflammatory, antidiabetic, antiobesity, antibacterial, antifungal, antinematode, and antiviral. Besides positive physiological properties, CC has also negative features, for example, skin reactions in patients with dermatitis and atopic predisposition, risk of confusion with poisonous mushrooms, quick autolysis after collection, and contamination of toxic elements.

## 1 INTRODUCTION

Mushrooms have been used as food and also as traditional medicine by their content of components with pro‐health activity (Demirbas, 2001; Gao, 2006). A great example of these mushrooms belonging to the phylum Basidiomycota is *Coprinus comatus* (O.F.Müll.) Pers., also called shaggy mane, chicken drumstick mushroom, or lawyer's wig, usually grows in spring and autumn on lawns. *C*. *comatus* (CC) belongs to the phylum Basidiomycota, family Agaricaceae, and *Coprinus* genus, which share name with it. Orton and Watling reported that in 1780 CC was categorized by Otto Friedrich Müller and first named *Agaricus comatus*. Seventeen years after, Christiaan Hendrik Persoon changed name *A*. *comatus* to CC and transferring this mushroom to *Coprinus* genus (Kirk, Cannon, Minter, & Stalpers, 2008; Orton & Watling, 1979).

**FIGURE 1:**
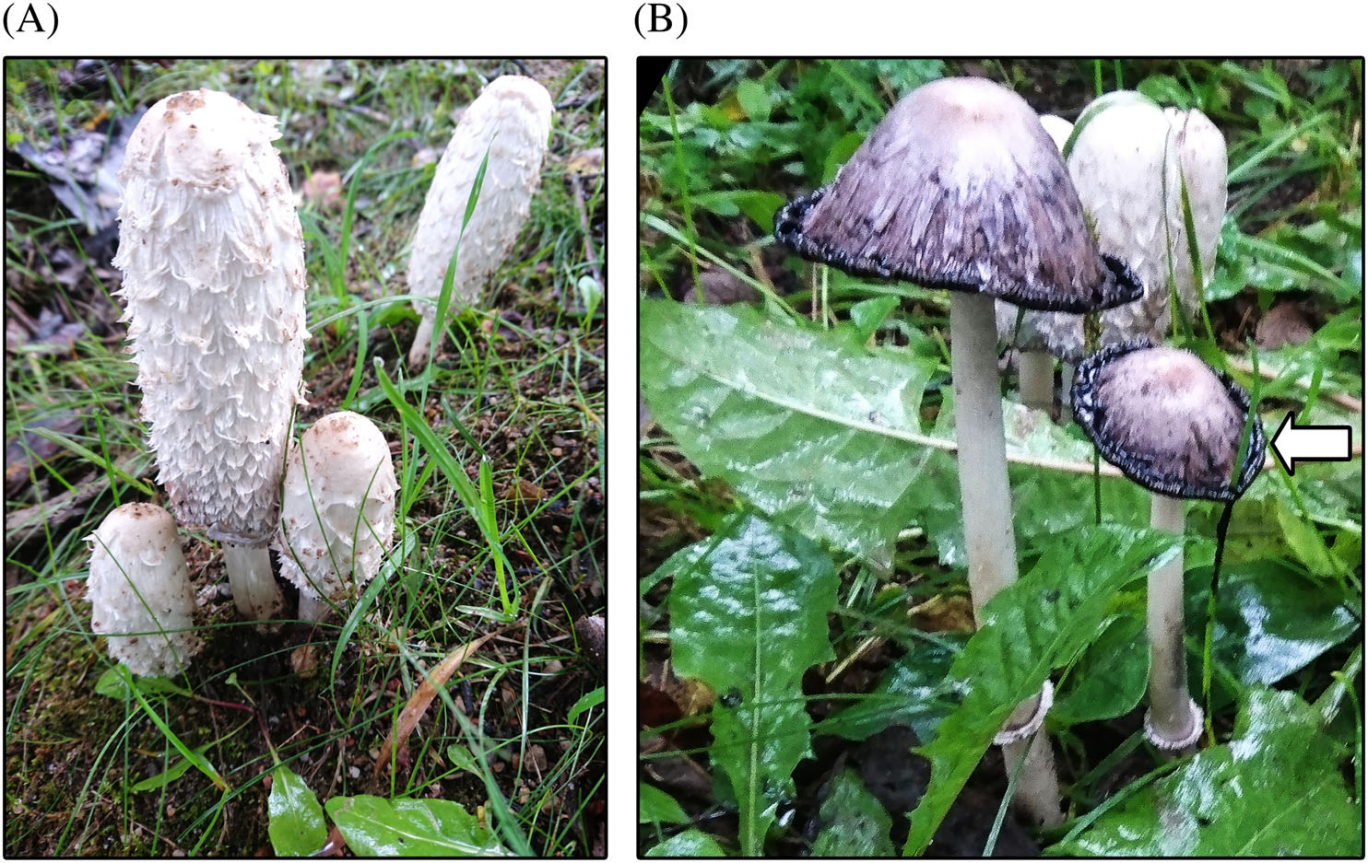
*Coprinus comatus* in the forest. (A) The young fruit bodies, at edible stage. (B) The old mushrooms beginning to autodigest and turn into a black inky liquid (arrow) [Colour figure can be viewed at wileyonlinelibrary.com]

The unique feature of this species is that it is edible only when young, old one undergoes autolysis. The cap of CC is normally white, but with time it turns pink and covers the stipe over (Figure 1). After depositing spores or being picked, it changes its color to black and dissolves itself in a matter of hours (Rouhana‐Toubi, Wasser, Agbarya, & Fares, 2013). Normally, a cap is from 5 to 10 cm tall, initially egg shaped, opens into a long bell. When it is white, its top breaks up into large recurved scales. The stem of CC is white, hollow, and 6–15 cm tall. The stem ring becomes colored with black spores. Spores are black, smooth, and ellipsoidal with size of 9–13 × 7–9.5 μm. It occurs in woods, meadows, and verges of roads. Many physiological effects of CC have been reported, for example, antioxidant, anticancer, antiandrogenic, hepatoprotective, acetylcholinesterase inhibitory, antiinflammatory, antidiabetic, antiobesity, antimicrobial, antiviral, antifungal, and antinematode activity (Dotan, Wasser, & Mahajna, 2011; Li, Lu, Suo, Nan, & Li, 2010; Park et al., 2014; Sabo et al., 2010; Stojković et al., 2013; Zaidman, Wasser, Nevo, & Mahajna, 2008; Zhang et al., 2017; S. Zhao et al., 2014; Zhou & Han, 2008). CC is valued for its taste as well as for nutritional properties; therefore, in 2006, in China, 382,000 tons of this mushroom have been consumed (Bailey, Turner, Jakeman, & Hayes, 1984; Fan et al., 2006). CC is cultivated as an edible mushroom in Japan, China, and other Asian countries, but in some countries in Europe, for example, and in Poland, it is not legally classified as an edible mushroom (Polish Journal of Laws, 2018). The aim of the presented work is to discuss the composition related to functional properties as well as the potential risks associated with consumption of CC by reviewing scientific literature.

## 2 NUTRITIONAL VALUE AND CHEMICAL COMPOSITION OF CC

Mushrooms are appreciated for their taste but also for nutritional value. Nutritional value of CC was examined by various researchers. The assays show that 100 g dry weight (d.w.) of CC provided energy in amount of 368.1–525 kcal. The main compounds of mushrooms were carbohydrates. The content of carbohydrates in CC was 49.2–76.3 g/100 g d.w. It is worth emphasizing that CC was a good source of dietary fiber because dried CC contained 32.8 ± 4.2% water‐insoluble and 1.79 ± 1.1% water‐soluble fiber. CC has been reported to have 11.8–29.5 g of protein and 1.1–5.4 g of fat in 100 g d.w. (Akata, Ergonul, & Kalyoncu, 2012; Cheung, 2013; Stojković et al., 2013; Vaz et al., 2011). Nutritional value of protein from mushrooms is related to ratio of various protein fractions. The analyses detected six different protein fractions: albumins, globulins, prolamines, prolamines‐like fraction soluble in alcohol after reduced with 2‐mercaptoethanol, glutelin‐like fraction soluble in alkali, and true glutelin. Protein fractions were presented as percentage of total protein in CC and reached value 14.75 ± 0.72%, 27.36 ± 0.65%, 5.48 ± 0.18%, 5.27 ± 0.26%, 4.48 ± 0.14%, and 6.97 ± 0.17%, respectively. Moreover, the total protein content in mushroom was higher than in other foods mentioned by Petrovska, such as barley, amaranth grain, maize, rice, wild rice, wheat, and sorghum (Petrovska, 2001). It is worth emphasizing that protein from mushroom like CC is also highly digestible, and usually it is in a range 71–90%. It was found that 2 g protein from mushroom is equal to 1 g meat protein. Therefore, in Eastern Europe mushrooms were sometimes called ’forest meat‘ or ’meat for poverty’ (Kalač, 2016; Mukerji & Manoharachary, 2010).

The chemical composition of CC fruiting body depends on origin, environmental condition, and so forth. Mushrooms are rich in various types of biologically active substances and their metabolites with many different properties (Tang, Yin, Zhang, Jia, & Gao, 2015). Some of these compounds were found only in cultivated mushrooms, whereas others only in wild ones (Table 1). Trehalose dominates in the carbohydrates group of free sugars (Stojković et al., 2013). Polysaccharides extracted from water extract of CC fruiting bodies were fractionated by size‐exclusion chromatography and analyzed by 1D/2D NMR spectroscopy. Detailed analysis has shown presents of disaccharide α, α‐trehalose [α‐d‐Glcp‐(1→1)‐α‐d‐Glcp], β‐d‐glucans containing of β‐d‐Glcp‐m, lower molecular mass penta‐saccharide‐repeating α‐l‐fuco‐α‐d‐galactan, (→6)‐α‐d‐Galp‐(1→6)‐[α‐l‐Fucp‐(1→2)‐]α‐d‐Galp‐(1→6)‐α‐d‐Galp‐(1→6)‐α‐d‐Galp‐(1→}p. The most common component was α‐d‐glucans containing of [→4)‐α‐d‐Glcp‐(1→]n backbones with roughly 10% of branching at C‐6 by terminal α‐d‐Glcp‐(1→6)‐ or α‐d‐Glcp‐(1→6)‐α‐d‐Glcp‐(1→6)‐ sequences (Li, Dobruchowska, Gerwig, Dijkhuizen, & Kamerling, 2013). The amino acids with the largest concentration were glutamic acid (441.6 mg/100 g d.w.) and alanine (222.8 mg/100 g d.w.). In addition, CC contained lower content of cys‐thionine (1.9 mg/100 g d.w.) and methionine (5.3 mg/100 g d.w.). Polyunsaturated fatty acids (PUFA) were the main fatty acids fraction in CC and reached value 66.01%. Saturated fatty acids (SFA) were 18.72%, and monounsaturated fatty acids (MUFA) were 15.27% (Stojković et al., 2013). Pedneault, Angers, Gosselin, and Tweddell also determined quantity of PUFA, SFA, and MUFA, and there were 65.3, 23.8, and 10.4%, respectively. Furthermore, fatty acids profiles of two fractions: neutral and polar lipids were determined. The both lipid fractions were chiefly composed of linoleic acid (18:2 Δ9c, 12c; 64.5% of neutral lipids and 63.7% of polar lipids) or palmitic acid (16:0; 18.9% of neutral lipids and 20.4% of polar lipids). The lower concentrations were measured in the fatty acids with small number of carbon and none of double bonds (Pedneault et al., 2008). Moreover, the content of the total tocopherols (588.2 μg/100 g) in cultivated CC is worth attention, because it is significantly higher than the total tocopherols level in wild CC (Stojković et al., 2013). An important component of mushrooms is provitamin D2 called ergosterol, but the content of ergosterol has not been determined in CC yet (Kalač, 2016; Reyes et al., 2009; Teichmann, Dutta, Staffas, & Jägerstad, 2007; Villares, Mateo‐Vivaracho, García‐Lafuente, & Guillamón, 2014).

**TABLE 1:** The content of selected chemical compounds in *Coprinus comatus* (Pedneault, Angers, Gosselin, & Tweddell, 2008; Reyes et al., 2009; Stojković et al., 2013)

Group of compounds	Compound	Content in 100 g d.w. (mean ± SD)
Free sugars (g)	Fructose	0.11 ± 0.1^a^
	Mannitol	1.84 ± 0.1
	Trehalose	5.41 ± 0.4
Amino acid (mg)	Aspartic acid Threonine Serine Asparagine Glutamic acid Glutamine Glycine Alanine Valine Cysteine Methionine Isoleucine Tyrosine Phenylalanine Histidine Lysine Tryptophan Arginine Proline Phosphoserine α‐amino adipic acid α‐aminobutyric acid Cys‐thionine γ‐aminobutyric acid Ornithine	70.6 61.7 76.2 39.6 441.6 57.4 55.2 222.8 94.4 22.3 5.3 63.0 80.6 61.2 59.6 31.0 64.8 18.2 57.5 60.0 112.5 6.4 1.9 41.9 36.1
Fatty acids profiles of neutral/polar lipids (%)	4:0 5:0 6:0	0.02/− 0.02/− 0.03/−
	7:0 8:0 9:0 10:0 11:0 12:0 13:0 14:0 14:1 Δ9c 15:0 16:0 16:1 Δ7c 16:1 Δ9c 16:1 Δ11c 16:2 Δ7c, 10c 16:2 Δ9c, 12c 17:0 17:1 Δ9c 18:0 18:1 Δ9t 18:1 Δ9c 18:1 Δ11c 18:2 Δ9c, 12c 18:3 Δ9c, 12c, 15c 20:0 20:1 Δ9c 20:1 Δ11c 20:2 Δ11c, 14c 21:0 22:0 22:1 Δ13c 23:0 23:1 Δ14c 24:0 24:1 Δ15c 25:0 26:0	0.03/− 0.02/− 0.04/− 0.02/0.04 0.05/0.02 0.13/0.12 0.03/0.04 0.57/0.78 0.03/0.04 0.37/0.44 18.9/20.4 0.09/0.3 0.87/1.14 0.7/0.56 0.07/− 0.07/0.07 0.08/0.32 0.05/0.04 1.84/1.98 0.12/0.1 7.45/5.88 0.65/0.71 64.5/63.7 0.47/1.07 0.13/0.15 0.04/− 0.18/0.43 0.35/0.25 0.08/0.08 0.33/0.18 0.05/0.07 0.14/0.08 0.02/− 0.89/0.75 0.17/0.13 0.06/0.05 0.04/0.06
	SFA (relative %)	18.72 ± 0.1
	MUFA (relative %)	15.27 ± 0.1
	PUFA (relative %)	66.01 ± 0.1
Organic acids (g)	Oxalic acid	0.68 ± 0.0
	Quinic acid	3.37 ± 0.4
	Malic acid	4.08 ± 0.2
	Citric acid	11.84 ± 0.2
	Fumaric acid	0.65 ± 0.0
Tocopherols (μg)	α‐Tocopherol	13.24 ± 0.7
	β‐Tocopherol	375.99 ± 10.8^a^
	γ‐Tocopherol	165.57 ± 6.7^a^
	δ‐Tocopherol	31.76 ± 2.0

Abbreviations: MUFA, monounsaturated fatty acids; PUFA, polyunsaturated fatty acids; SFA, saturated fatty acids.

^a^ Detected in cultivated but not in wild mushrooms.

Tešanović et al. detected polyphenol compounds, group of the secondary metabolites with bioactivity, such as flavones, flavonols, flavanones, flavanols, biflavonoids, isoflavonoids, hydroxybenzoic acids, hydroxycinnamic acids, coumarins, and chlorogenic acids in CC water extract (Table 2). Among phenolic compounds, the largest content was detected for quinic acid (14.6 mg/100 g d.w.) and quercetin (3.01 mg/100 g d.w.). Whereas the lowest amount was detected for isoflavonoids: genistein (0.023 mg/100 g d.w.) and daidzein (0.061 mg/100 g d.w.) (Tešanović et al., 2017). The majority of polyphenols existed as aglycone or glycoside forms, which affects biological property. Glycosylation of polyphenols decreased bioactivity, like antibacterial, antioxidant, antidiabetic, antiinflammatory, and anticancer, although it enhance antistress, antiobesity, antirotavirus, and antiallergic activity (Ng & See, 2019; Xiao, 2017). CC extracts can also contain other compounds with health benefits, for example, laccase, glycan binding protein, and triglycerides (Ren, Shi, Han, Liu, & Guo, 2012; Zhang et al., 2017; Zhao et al., 2014).

**TABLE 2:** The content of phenolic compounds in *C*. *comatus* aqueous extract (Tešanović et al., 2017)

Groups of compounds	Compound	Content (mg/100 g d.w.)
Flavones	Apigenin	0.141
	Baicalein	0.544
	Chrysoeriol	0.143
	Vitexin	0.193
	Apigenin‐7‐O‐glucoside	0.201
	Luteolin‐7‐O‐glucoside	0.070
	Apiin	0.170
	Baicalin	0.898
Flavonols	Quercetin	3.010
	Isorhamnetin	0.582
	Quercitrin	0.108
	Kaempferol‐3‐O‐glucoside	0.182
	Hyperoside	0.026
	Quercetin‐3‐O‐glucoside	0.105
	Rutin	0.146
Flavanones	Naringenin	0.259
Flavanols	Catechin	0.454
	Epicatechin	0.336
Biflavonoids	Amentoflavone	0.484
Isoflavonoids	Daidzein	0.061
	Genistein	0.023
Hydroxybenzoic acids	*p*‐Hydroxybenzoic acid	0.928
	Protocatechuic acid	0.480
	Syringic acid	0.356
Hydroxycinnamic acids	*p*‐Coumaric acid	0.185
	*o*‐Coumaric acid	0.116
	Caffeic acid	0.158
	Ferulic acid	0.149
Coumarins	Esculetin	0.148
	Scopoletin	0.197
	Umbeliferon	0.160
Chlorogenic acids	Quinic acid	14.600
	5‐O‐Caffeoylquinic acid	0.554

Mushrooms contain many essential minerals. Tel et al. quantified composition of microelements and macroelements in CC and other wild mushrooms. Macroelements dominated in CC were phosphorus (5,726.4 mg/kg d.w.), potassium (4,077.2 mg/kg d.w.), magnesium (1,348.5 mg/kg d.w.), sodium (291.7 mg/kg d.w.), and calcium (157.2 mg/kg d.w.). The microelements contained in CC were iron (237.9 mg/kg d.w.), zinc (53.25 mg/kg d.w.), and manganese (10.97 mg/kg d.w.) (Tel et al., 2014).

Composition, nutritional value, and also potentially pollution of CC may depend on growing condition. Jang et al. tested optimal conditions and growing medium to cultivate CC. The results show that the best growing medium was that contained agar, peptone, malt, and yeast extracts. Favorable mycelial growth was in temperature 26°C and pH 7. The most effective source of carbon was sucrose and nitrogen source was tryptone (Jang, Lee, Liu, & Ju, 2009).

## 3 THE POSITIVE INFLUENCE OF CC ON THE HUMAN HEALTH

### 3.1 Antioxidant activity

Various mushrooms including CC have been reported as therapy support in many human diseases because of a large range of activities on human body. One of them is the antioxidant activity which is closely related to other pro‐health properties like anticancer, antiinflammatory, and antiobesity. Li et al. showed antioxidant properties of the stipe and cap from CC. Assay measured ability to inhibit linoleic acid peroxidation indicated that ethanol solution reached antioxidant activity level of about 80.6% at 1 mg/mL (extract from stipe of CC) and 70.5% at 5 mg/mL (cap of CC). However, antioxidant activity of water extract from stipe was 61.5% and from cap 72.6% in higher concentration—10 mg/mL. Moreover, antioxidant activity of ethanol solution extracts from cap was more intense than that of l‐ascorbic acid at 1 mg/mL concentration (Li et al., 2010). In another study, antioxidant effect of ethanol solution extracts from CC at 1 mg/mL concentration reached value 63.4%. However, the water extracts from fruiting body of CC reached 65.6% at concentration 5 mg/mL (Tsai, Tsai, & Mau, 2009).

The reducing power of CC extracts was also tested with spectrophotometric method, which measures the force to reduce ferricyanide to ferrocyanide. The reducing power of ethanol solution extract of cap and stipe at 10 mg/mL concentration was 1.653 and 0.364, while water extracts reached 0.998 and 1.122, respectively (Li et al., 2010). The reducing power of CC ethanol solution extract in a different studies was 0.50 at 10 mg/mL concentration and in water extract was 0.48 at 5 mg/mL (Tsai et al., 2009). However, reference substance, which was l‐ascorbic acid, showed reducing power of 2.087 at 1.0 mg/mL (Li et al., 2010). Naturally occurring antioxidant components, such as ascorbic acid, β‐Carotene, lycopene, and various phenolic compounds were found in the CC extracts (Sánchez, 2016). Ethanol solution extracts contained more flavonoids and tocopherols but less polysaccharides than hot water extracts. The authors showed differences in the chemical composition and the antioxidant activity between the cap and stipe. Overall, CC extracts from cap were better antioxidant than stipe extracts (Li et al., 2010). Ren et al. tested impact of CC on total antioxidant status (TAOS), assay which indirect measured formation various oxidant species. The triglycerides from the fermented CC (TFC) reduce level of TAOS in every tested concentration compared with control. Ren et al. claims that the reduction of TAOS level causing antiinflammatory effect of TFC (Ren et al., 2012).

Similar results were published by Cao et al., who tested the antioxidant and hypoglycemic effects of polysaccharides fractions of fermented CC called extracellular polysaccharides (ECPs), intracellular polysaccharides (ICPs), deproteinized extracellular polysaccharides (EDPs) and deproteinized intracellular polysaccharides (IDPs). The highest DPPH (α‐diphenyl‐β‐picrylhydrazyl) radical scavenging capacity from of all tested CC fractions was ICPs (63.78 ± 0.38%) in 10 mg/mL concentration. The highest OH radical scavenging capacity in 10 mg/mL concentration reached ICPs with 91.85 ± 1.38 U/mL and ECPs with 84.95 ± 0.78 U/mL (Cao et al., 2019).

Data presented in Table 3 shows antioxidant properties, like scavenging ability on DPPH, OH and superoxide radicals in ethanol solution and water extracts from cap and stipe. Scavenging ability on DPPH and OH radicals were the highest in ethanol solution extracts from CC cap (0.86 mg/mL and 3.23 mg/mL, respectively) and scavenging ability on superoxide radicals was the highest in ethanol solution extracts from stipe (20.7 mg/mL; Li et al., 2010).

**TABLE 3:** EC_50_ (half maximal effective concentration) values of ethanol solution and hot water extracts from cap and stipe of *Coprinus comatus* in antioxidant properties (Li et al., 2010)

Antioxidant attribute	Cap ‐ EC_50_ (mg/mL)		Stipe ‐ EC_50_ (mg/mL)	
	Ethanol solution	Hot water	Ethanol solution	Hot water
Antioxidant activity	1.56 ± 0.24	0.81 ± 0.03	0.62 ± 0.42	5.08 ± 0.17
Reducing power	1.67 ± 0.13	0.95 ± 0.05	1.93 ± 0.23	14.8 ± 0.22
Scavenging ability on DPPH radicals	0.86 ± 0.06	2.09 ± 0.26	7.86 ± 0.16	8.98 ± 0.19
Scavenging ability on OH radicals	3.23 ± 0.28	8.66 ± 0.35	3.35 ± 0.17	16.9 ± 0.41
Scavenging ability on superoxide radicals	25.3 ± 0.21	No effect	20.7 ± 0.38	No effect

Abbreviation: DPPH, α‐diphenyl‐β‐picrylhydrazyl.

Polysaccharides from CC affects on hepatic and mitochondrial antioxidant enzymes, such as glutathione peroxidase (GSH‐Px), superoxide dismutase (SOD) and catalase (CAT). Treatment with polysaccharides from CC increased activity of hepatic GSH‐Px about 166.78%, SOD about 83.72% and CAT about 63.12%. However, activity of mitochondrial enzymes GSH‐Px, SOD and CAT was increased by CC about 92.00%, 67.03%, and 51.61%, respectively (Zhao et al., 2019). Song & Du proved medium antioxidant ability of polysaccharides from CC compared with other tested mushrooms on assays: superoxide anion radical scavenging activity (SRSA), reducing power (RP), chelating ability (CA) and weak antioxidant ability on assays: hydroxyl radical scavenging activity (HRSA) and DPPH scavenging ability (DSA) (Song & Du, 2011). Hydroxo perhydroxo mercury(II) complex assay (HPMC) performed by Karaman et al. showed antioxidant property of 3 extracts from CC. Fruiting body extract (FBE) of CC reached antioxidant activity value 2.3 ± 0.1%/μL, mycelia extract (ME) reached 1.7 ± 0.1%/μL and activity of filtrate extract (FE) was 2.0 ± 0.1%/μL. Moreover, content of quinic acid was evaluated and it was also higher in FBE (46.1 mg/g d.w.) compared with ME (1.3 mg/g d.w.) and FE (1.9 mg/g d.w.) (Karaman et al., 2019).

Numerous reports of CC health‐promoting properties led to the creation of a commercial preparation of CC. Popović et al. shows the effects of CC aqueous suspension on the expression of antioxidant markers in homogenate from rat liver. The study reported that CC significant increased level of glutation (GSH) compared with control samples (Popović, Vukmirović, Stilinović, Capo, & Jakovljević, 2010). GSH is non‐protein thiol, which participates in ability to scavenging reactive oxygen species (Coco‐Bassey et al., 2019). One week therapy with CC before dose of carbon tetrachloride resulted in a significant increase of xanthine oxidase (XOD), lipid peroxidation and peroxidase. Carbon tetrachloride was prooxidant factor which also increase the intensity of peroxidation in lipid. Furthermore, Popović, Vukmirović, Stilinović, Capo, & Jakovljević claims that CC has ability to protect against carbon tetrachloride toxicity because GSH level in group treater CC with carbon tetrachloride was almost equal to treated only CC group (Popović et al., 2010).

### 3.2 Antiinflammatory effects

Ren et al. demonstrated analgesic and antiinflammatory effects of TFC. The inflammatory response is linked to a signal promoting kinase release. In acute inflammation induced by carrageenan in mice, therapy with triglycerides from CC in a dose of 30 mg/kg body weight (b.w.) reduced proinflammatory factors: tumor necrosis factor α (TNF‐α) by 58%, interleukin 1 beta (IL‐1β) by 27%, vascular endothelial growth factor alpha by 47%, and interleukin 17 (IL‐17) by 89%. The writhing test, in which abdominal constrictions are induced by acetic acid, was used to screen analgesic and antiinflammatory effect of TFC. TFC in a dose‐dependent manner inhibited abdominal constrictions through peripheral antinociceptive activity. Although, TFC did not show central antinociceptive properties measured by the hot‐plate test (usually used to assess analgesic effect of narcotic and other drugs) (Calcagni & Elenkov, 2006; Ren et al., 2012). Polysaccharides from CC significant attenuated level of interleukin 6 (IL‐6), inducible nitric oxide synthase (iNOS), and cyclooxygenase 2 (COX‐2) indicated that CC reduce inflammatory response caused by alcohol (Zhao et al., 2019). The writhing test that evaluates analgesic property showed that extracts from fermented CC at 1 and 5 mg/kg concentration inhibited analgesic activity by 19 and 21%, respectively. Antinociceptive effect was tested by the formalin test and reaches inhibition value 3% at first phase and 6% at second phase at 10 mg/kg concentration (Han, 2009).

Zhao et al. examined influence of flavones from coculture broth of CC and *Morchella esculenta* on macrophages RAW264.7 stimulated by lipopolysaccharide. The results show that the flavones inhibited productions of pro‐inflammatory NO in dose‐dependent manner. The tested flavones also inhibits productions of other inflammatory mediator like TNF‐α, IL‐1β, iNOS, and COX‐2. Moreover, CC affects mitogen‐activated protein kinase (MAPK) signaling pathways by inhibition of serine/threonine kinase 1, N‐terminal protein kinase 1 and 2, and p38 expression (Zhao et al., 2018). MAPK signaling pathways are related with inflammatory activated by macrophages (Kaminska, 2005). Asahi et al. examined impact of CC extracts and ergothioneine isolated from CC on myeloperoxidase (MPO) activity. Leukocytes secreted protein MPO which generate inflammation and play role in progression of disease through releasing hypobromous acid and hypochlorous acid. Extract of CC and ergothioneine inhibited activity of MPO already at 1 μM concentration in dose‐dependent manner. Ergothioneine from CC at 100 μM concentration reduced activity of MPO to 0%. Moreover, extract from CC showed the same effect at 1 mg/mL concentration. CC also strongly decreased (almost 100%) activity rate of inflammatory marker 8‐bromo‐2′‐deoxyguanosine (8‐BrdG) at 1,000 μM concentration of ergothioneine and 1 mg/mL concentration of CC extract (Asahi et al., 2016; Gaut et al., 2001). These results indicated antiinflammatory properties of CC.

### 3.3 Anticancer potential

CC extract can modulate viability and proliferation of cancer cells. Zaidman et al. proved that ethyl acetate extract from CC inhibited proliferation of androgen‐sensitive human prostate adenocarcinoma cells LNCaP, through decreasing transcriptional activity of androgen receptors (AR). *Coprinus comatus* extract decreased activity of luciferase—enzyme which reveals AR transcriptional equal to the level of prostate‐specific antigen (PSA), which is a glycoprotein marker used for staging and screening of prostate cancer. The treatment with CC ethyl acetate extract inhibited PSA level by 77% (Zaidman et al., 2008). Dotan, Wasser, and Mahajna indicated that hexane extract showed the strongest antiandrogenic effect compared with ethyl acetate, chloroform, or ethanol extracts from CC (Dotan et al., 2011). The extract decreased PSA mRNA and AR protein level in LNCaP cells, inhibited colony formation in LNCaP cells and AR transcription activity in MDA‐kb2 cells. The study presented CC as an antiandrogenic modulator that could improve treatment of prostate diseases.

The recent studies proved an effect of CC against human T‐cell leukemia. Moreover, glycan‐binding protein isolated from CC called Y3 showed also anti‐Tobacco mosaic virus property. The analysis indicated that Y3 is an 18‐aa signal peptide with n‐terminus and n‐glycosylation site. Zhang et al. confirmed that Y3 had the effect on growth inhibition and caused induction of caspase‐dependent apoptosis in Jurkat cells of human T‐cell leukemia. Assays using 7‐aminoactinomycin D and Annexin V double staining indicated induction dose‐dependent manner effect of Y3 on early and also late apoptosis of Jurkat cells (90% apoptotic cells of total cells). In this study, Y3 shows only weak effect on cells viability against cervical cancer HeLa cells, pancreas carcinoma Dan‐G cells, and liver carcinoma HepG2 cells (Zhang et al., 2017).

accase from mycelia of CC may have antiproliferative properties. In nature, laccase takes a part in various physiological processes, because of its important role in lignin degradation (Baldrian, 2006; Brijwani, Rigdon, & Vadlani, 2010). CC laccase N‐terminal amino acid sequence is AIGPVADLKV. The results from MTT assay confirmed suppressor effect against proliferation human liver cancer cells and breast cancer cells (MCF7) lines with IC_50_ values of 3.46 and 4.95 μM, respectively (Zhao et al., 2014). Asatiani et al. results performed on the MCF7 cell line showed that IC_50_ of CC extract was 76 ± 1.41 μg/mL and ethyl acetate extract was 32 ± 0.71 μg/mL. Anticancer effect of CC was caused by inhibition of inhibitor of kappa B (IκBα) phosphorylation what lead to induction of the nuclear factor kappa‐light‐chain‐enhancer of activated B cells (NF‐κB) pathway in dose‐dependent manner (Asatiani et al., 2011).

Emsen and Guven proves that methanol and aqueous extracts of CC were bereft of genotoxicity in human lymphocytes cells, despite the anticancer effect of CC. In addition, CC did not show an effect on human lymphocytes proliferation. In the tested cells, oxidative stress level was inhibited by high concentration of CC and it could be linked to increase of the capacity of total antioxidant in cells with CC extracts (Emsen & Guven, 2019).

### 3.4 Hepatoprotective activity

Polysaccharides from CC have been reported as biologically active which may induce liver recovery after damage caused by alcohol consumption. This health and social problems are correlated with liver illness, for example, hepatitis, cirrhosis, and fatty liver (Yuan, Gong, Li, & Li, 2007). In animal study, Ozalp et al. indicated that treatment with CC polysaccharides extract in a dose of 50 mg/kg b.w. may repair liver damage caused by alcohol (Ozalp et al., 2014).

### 3.5 Acetylcholinesterase inhibitory property

Extract from CC was also screened for acetylcholinesterase (AChE) inhibitory potential. AChE takes a part in a synthesis of acetylcholine neurotransmitter (Basiri et al., 2013; Giacobini, 2004). Progressive cognitive impairment in Alzheimer's disease is connected with neurotransmitter acetylcholine deficiency and synaptic failure (Bartus, 2000). Inhibitors of cholinesterase stimulate the cholinergic receptors, increase availability of acetylocholine in the synaptic cleft, and weaken Alzheimer's disease symptoms (Anand, Patience, Sharma, & Khurana, 2017). The extract from CC had AChE inhibitory potential and reached IC_50_ value of 0.62 mg/mL. The results of the study emphasize the possibility of using CC extracts in the palliative therapy of Alzheimer's disease (Pejin et al., 2019).

### 3.6 Antidiabetic properties

Many studies from around the world confirmed the hypoglycemic effect of CC (Lv, Han, Yuan, & Guo, 2009; Yu et al., 2009; Zhou & Han, 2008). Zhou and Han tested the potential influence of combination of CC and vanadium on glycemic metabolism. In this study, homogenized fermented CC fruiting body was used in culture medium containing sodium metavanadate—NaVO3 (CCRV). The level of hepatic glycogen was increased by the use of aforementioned combination. In mice fed on CCRV, glycogen level was at 27.6 ± 5.2 mg/g, and it was higher compared with diabetic mice with value of 14.1 ± 3.8 mg/g and value 24.1 ± 4.3 mg/g in normal mice. Damaged pancreatic β‐cells were easily perceptible in diabetic mice. CCRV‐fed mice did not show loss of pancreatic cells. The islet cells of mice treated with CCRV were partially regenerated. The results showed antidiabetic activity of CCRV through reduction of hyperglycemia in diabetic mice, inhibiting gluconeogenesis, increasing glycogen, increasing insulin, and regenerating of injured β‐cells. Polysaccharides from CC have hypoglycemic activity because of inhibition of nonenzymatic glycosylation (NEG), which leads to attenuate increases in concentration of blood glucose (Zhou & Han, 2008). Han et al. showed effects of CCRV on glucose level in blood in alloxan treated mice. CCRV reduced level of glucose (10.5 ± 2.0 mmol/L) compared with alloxan‐treated mice (21.2 ± 2.1 mmol/L). Also, level of blood glucose at 60th minute in CCRV treated mice with hyperglycemia induced by adrenaline was reduced (10.6 ± 1.5 mmol/L) compared with adrenaline‐hyperglycemic mice (15.1 ± 1.0 mmol/L). Moreover, CCRV decreased glycosylated hemoglobin A1c (HbA1c) concentration (7.9 ± 0.28%) compare with the control (10.8 ± 0.23%; Han, Yuan, Wang, & Li, 2006). HbA1c is parameter that measure hyperglycemia and risk of complications of diabetes (Sherwani, Khan, Ekhzaimy, Masood, & Sakharkar, 2016).

Cao et al. examined CC impact on activity of α‐amylase. This enzyme hydrolyzed glycosidic bonds and it is responsible for activity of enzymes in digestive track and saliva, absorption of carbohydrates, and control postprandial level of glucose in blood. All of that affects a progress of diabetes (Bhandari, Jong‐Anurakkun, Hong, & Kawabata, 2008). The inhibition of α‐amylase causes reduced level of glucose and also reduced postprandial glucose (Ng & Rosman, 2019). The all polysaccharides extracts of CC tested by Cao et al. inhibit activity of α‐amylase at 2–10 mg/mL concentration. Extract called ICPs had the highest inhibitory effect on α‐amylase and reached 87.15 ± 0.99% (Cao et al., 2019). Polysaccharides from CC caused inhibition of nonenzymatic glycosylation that can limit diabetic complications, like macroangiopathy and microangiopathy. CC polysaccharides extracts inhibited 98% of NEG level at ≥30 mg/mL concentration. The chart of CC inhibitory effect on NEG reached almost similar value like antidiabetic drug—metformin—but was more sharp (Ding, Wang, Wang, Wang, & Zhang, 2012).

Comatin, compound isolate from CC, also showed antidiabetic properties. Ding et al. compared hypoglycemic effect of comatin with popular antidiabetic drug metformin. Comatin decreased a level of glucose in blood more than metformin after 1, 2, 3, and 4 hr in alloxan‐induced diabetic rats. Furthermore, comatin stronger than metformin reduced glycometabolism and lipometabolism parameters like fasted blood glucose (40.7% compared with 21.7%), postprandial blood glucose (49.8% compared with 22.8%), fructosamine (23.4% compared with 16.6%), total cholesterol (49.3% compared with 29.7%), and total triglycerides (28.7% compared with 19.1%) (Ding et al., 2010).

### 3.7 Antiobesity effect

*Coprinus comatus* has also antiobesity effect and plays a role in adipogenesis. The results based on differentiation of preadipocytes into adipocytes show that CC inhibits intracellular TG 3T3‐L1 adipocytes and reduces the content of triglycerides by 21% at 40 mg/mL concentration and 43% at 150 mg/mL. Increasing size and number of adipocytes correlate with higher lipid deposition. The main regulator of adipocyte gene expression and adipocyte differentiation is peroxisome proliferator‐activated receptor gamma (PPARγ). Activation of PPARγ causes lipoprotein lipase expression, adipocyte protein 2, as well as adiponectin and fatty acid synthase (Gregoire, Smas, & Sul, 1998). The phosphatidylinositol 3‐kinase/Akt signaling pathway is a second path of adipogenesis regulation and adipocyte differentiation (Magun et al., 1996; Sakaue et al., 1998). The MTT assay showed that CC did not affect on viability of 3T3‐L1 adipocytes cells at up to 150 μg/mL concentration. The treatment with CC reduced mRNA levels of PPARγ and C/EBPβ (CCAAT‐enhancer‐binding proteins) in a time‐ and concentration‐dependent manner. CC prevented adipocyte differentiation because of its antagonistic effect on PPARγ. Assays demonstrated that 150 μg/mL of CC extract significantly downregulated expression of C/EBPβ and PPARγ in comparison to the control. CC extract took a part in Akt/GSK3β pathway regulation of adipocyte differentiation. Extract in 150 μg/mL concentration reduced insulin‐stimulated uptake of glucose in adipocytes by 35%. The extract reduced fat mass and body weight what was confirmed in obese rats. After 5 weeks of treatment with CC extract, the body weight was substantially reduced by 25% at 60 mg CC extract/kg b.w. and 36% at 200 mg/kg b.w., compared with the control group. Therapy using 200 mg/kg b.w. CC extract reduced total triglycerides and total cholesterol level in serum by 32 and 46%, respectively. Moreover, the high‐density lipoprotein level was significantly increased in the group treated with CC compared with the control. Expression of adipogenesis genes was also inhibited by the extract in high fat diet (HF‐diet) induced obese rats (Park et al., 2014).

### 3.8 Antimicrobial activity

The antimicrobial compound (3R,4S)‐2‐methylene‐3,4‐dihydroxypentanoic acid 1,4‐lactone was isolated from CC by De Carvalho et al. The CC lactone disrupted with quorum sensing and distracted biofilms of *Pseudomonas aeruginosa*, which also limited the formation of rhamnolipid B and pyocyanin pathogenicity factors (De Carvalho et al., 2016). Bacterial biofilm is thin sheet coating bacteria responsible for resistance to antibiotics and phagocytosis (Magun et al., 1996). Additionally, that compound also works against *Staphylococcus aureus* biofilms because it dispersed it at subtoxic level and cause inhibition of enzyme important for synthesis of cells wall which is UDP‐acetyl glucosamine enolpyruvyl transferase (De Carvalho et al., 2016). The assay performed by Kalaw and Albinto evaluated impact of acetone and ethanol extracts from CC on bacteria Gram (+) *S*. *aureus* after 24 hr incubation. The results reveled that ethanol and acetone extract from CC inhibited growth of *S*. *aureus* with 14.09 ± 4.65 and 13.16 ± 3.39 mm zone of inhibition, respectively (Kalaw & Albinto, 2014). Modi, Parihar, Pithawala, and Jain tested methanolic and aqueous extracts of CC on other bacterial cultures, such as *Salmonella typhi* MTCC‐733, *Escherichia coli* MTCC‐425, and *Bacillus cereus* MTCC‐430. Methanol extracts of CC reach inhibition zone value of 16 ± 0.5, 21 ± 0.7, and 14 ± 0.11 mm in *S*. *typhi*, *E*. *coli*, and *B*. *cereus*, respectively. Furthermore, aqueous extracts of CC reach value of 13 ± 0.5, 16 ± 1.0, and 20 ± 1.75 mm in *S*. *typhi*, *E*. *coli*, and *B*. *cereus*, respectively. The results were compared with inhibition zone of positive control (Streptomycin), which reached 27, 26, and 24 mm at bacterial cultures, respectively (Modi, Parihar, Pithawala, & Jain, 2014). Many others studies from all over the world confirmed antimicrobial activity of CC (Mwita, Mshandete, & Lyantagaye, 2010).

### 3.9 Antifungal

In many studies, the assay of antifungal properties was performed inter alia toward mycelium of CC (Ye & Ng, 2001; Ye, Ng, Tsang, & Wang, 2001). In addition to this, CC has its own antifungal activity confirmed on many microfungi species. The antifungal bioassay carried out by Stojković et al. indicated that methanolic extracts from cultivated CC shows the strongest effect toward *Trichoderma viride* minimum inhibitory concentration (MIC)—0.2 mg/mL—and minimum fungicidal concentration (MFC)—1.5 mg/mL and *Aspergillus versicolor* (MIC—0.2 mg/mL and MFC—1.5 mg/mL). In contrast, the weakest antifungal activity was achieved against *Penicillium verrucosum* var *cyclopium* and *Aspergillus fumigatus* (MIC—3.0 mg/mL and MFC—6.25 mg/mL). The effect toward *Penicillium ochrochloron* (MIC—0.2 mg/mL and MFC—3.0 mg/mL) was more efficient than treatment with ketoconazole (MIC—2.5 mg/mL and MFC—3.5 mg/mL) standard antifungal drug. The MIC value of CC against *A*. *versicolor*, *T*. *viride*, and *Penicillium funiculosum* (0.2 mg/mL) was lower or equal compared with ketoconazole (0.2, 1.0, and 2.5 mg/mL, respectively) (Stojković et al., 2013). The results of assessment performed by Florianowicz presented that water extract of CC decrease growth of *Penicillium expansum* mycelium. The inhibition range of CC was 9–13 mm compared with 15–18 mm of control (sample without extract), after 3 days of incubation. Whereas after 5, 7, and 9 days, ranges were 14–19, 19–24, and 20–25 mm compared with 20–22, 24–27, and 25–27 mm of control, respectively (Florianowicz, 2000).

### 3.10 Antinematode activity

*Coprinus comatus* has also proofed toxicity effect against nematode (Li & Xiang, 2005). Luo et al. observed on the vegetative hyphae of CC an exceptional structure called spiny ball. Further research on these structures shows that isolated and washed spiny balls were immobilized *Panagrellus redivivus* nematode. High‐resolution MS assay identified seven compound with nematotoxicity effect such as 5‐Methylfuran‐3‐carboxylic acid, 5‐Hydroxy‐3,5‐dimethylfuran‐2(5H)‐one, 5‐Hydroxy‐3‐(hydroxymethyl)‐ 5‐methylfuran‐2(5H)‐one, 4,6‐Dihydroxyisobenzofuran‐1,3‐dione, 4,6‐Dihydroxybenzofuran‐3(2H)‐one, 4,6‐Dimethoxyisobenzofuran‐1(3H)‐one, and 3‐Formyl‐2,5‐dihydroxybenzyl acetate. Mechanism of action was presented using scanning electron microscopy. Spiny balls were devastate cuticles of nematode, which cause outflow of internal nematode materials. First examined strain from CC cause immobilized 75.0 and 93.8% nematodes after 5 and 10 min exposure, respectively. Although, second strain immobilized 76.9 and 92.3% of nematodes after 5 and 10 min after being added on blank water agar plates with *P*. *redivivus* (Luo et al., 2007).

### 3.11 Antiviral

Purified laccase from CC may also cause inhibition of protein human immunodeficiency virus 1 reverse transcriptase (HIV‐1 RT) at IC_50_ = 5.85 μM. HIV‐1 RT is an important part in the cycle of retrovirus life because it takes part in synthesis of double‐stranded DNA from single RNA genome. Consequently, HIV‐1 RT was suggested as a main antiviral drug target and in the future it can be used in support of AIDS treatment (Das & Arnold, 2013; Zhao et al., 2014).

## 4 THE ADVERSE EFFECTS OF CC CONSUMPTION

### 4.1 Skin reactions

*Coprinus comatus* may cause skin reactions in patients with atopic dermatitis and atopic predisposition. The study of Fischer, Yawalkar, Brander, Pichler, and Helbling showed that in 32% patients with atopic dermatitis CC induced delayed‐type reactions after atopic patch test (containing 5 mg protein from cap per 1 g vaseline or 1.35 mg spore protein per 1 g vaseline). After 48 and 72 hr, negative skin test reaction was observed in the nonatopic control group. Immunohistochemical and histologic analyses show that the reaction was consistent with acute skin changes in atopic dermatitis. Reaction for CC was specific T‐cell response because of dominance of CD4+ cells (Fischer, Yawalkar, Brander, Pichler, & Helbling, 1999).

### 4.2 The risk of confusion with poisonous mushrooms

*Coprinus comatus* can sometimes be confused, mainly with the *Coprinopsis atramentaria* (Bull.) Redhead, Vilgalys, and Moncalvo. *C atramentaria* is found in places and has a cap similar to those of CC. *Coprinopsis atramentaria* is edible, but contains coprine, which causes negative disulfiram‐like reaction related to consuming this Basidiomycota with ethanol. Based on chemical analysis, coprine is N5‐(1‐hydroxycyclopropyl) glutamine (Hatfield & Schaumberg, 1975). Coprine causes inhibition of the dehydrogenase in the liver, which increases the level of acetaldehyde in the blood after alcohol consumption. However, in opposite to disulfiram‐like reaction, coprine does not inhibit dopamine‐beta hydroxylase. Disulfiram‐like reaction ensues if ethanol is consumed in time of 30 min to 3 days after the mushroom ingestion. It can happen within several minutes after consuming alcohol (Haberl, Pfab, Berndt, Greifenhagen, & Zilker, 2011; Köppel, 1993).

During alcohol metabolism, coprine blocks conversion of acetaldehyde into acetate and causes aldehyde accumulation in the liver and then in the blood (Tottmar & Lindberg, 1977). Usually, accumulation of acetaldehyde causes unpleasant effect, such as flushing, vomiting, nausea, tachycardia, headache, dizziness, hypotension, palpitations, and dyspnea (Haberl et al., 2011). Therapy of this reaction is supportive and consists of fluid and electrolyte replacement. Normally, the symptoms resolve after 6 hr from the time of alcohol ingestion and there is a risk that this reaction may recur after repeated alcohol consumption indicates that CC does not contain coprine (Berger & Guss, 2005; Berger & Guss, 2005a; Carisson et al., 1978; Diaz, 2005; Michelot, 1992; Peredy, 2014).

### 4.3 Only the young CC fruit body are edible

The majority of edible mushrooms can be eaten in all stages of their growth. However, CC is edible only when it is young, the old mushroom becomes inedible. At maturity, spores and hymenia transform through the process of autodigestion into inky, black fluid which makes CC darker (Figure 1b). The process of CC autodigestion reduces its culinary properties and nutritional values (Saiz‐Jimenez, 1983). CC fruiting bodies are highly perishable and must be consumed, processed, or iced within 4–6 hr of collection. Within 2 days, they undergo autolysis when they are stored after collection (Sas‐Golak, Siwulski, Sobieralski, & Lisiec, 2012).

### 4.4 Pollution of CC

Scientific studies confirm that mushrooms, for example, *Agaricus bisporus*, *Pleurotus ostreatus*, CC, and others could be used as a bioindicator of soils pollution with toxic metals due to the ability to absorb a large amount of its (García‐Delgado, Alonso‐Izquierdo, González‐Izquierdo, Yunta, & Eymar, 2017; Li et al., 2017). Researchers from across Europe show many data about high concentration of mercury (Hg) in CC. Total Hg concentration in CC from Germany (in mining area) was 144 mg/kg d.w., from Finland was 5.6 mg/kg d.w., Switzerland was 3.3 mg/kg d.w., and Slovenia was 2.1 mg/kg d.w. (Byrne, Ravnik, & Kosta, 1976; Fischer, Rapsomanikis, Andreae, & Baldi, 1995; Laaksovirta & Lodenius, 1979; Quinche & Dvorak, 1975). The content of Hg in CC from some sites in Poland was also high and reached median value 9.2 mg/kg d.w. in caps and 5.2 mg/kg d.w. in stipes (Falandysz, 2016). Eating contaminated mushrooms can expose consumer to relatively high dose of Hg. Based on the WHO norms, Provisional Tolerable Weekly Intake (PTWI = 4 μg/kg b.w.) and the Hg content in CC, the toxicity dose can be determined. It is estimated that the value that will exceed PTWI was from 20 g to 1.6 kg CC depending on the mushroom habitat (FAO, 2011). Mercury is definitely one of the most toxic elements to humans. All its forms alter physiological cellular function because it changes the structure of protein by binding with selenohydryl and sulfhydryl groups. The main target of Hg is the brain and also immune, endocrine, nerve, renal, and muscle functions (Berlin, 2003).

Mushrooms not only absorb Hg but they can also accumulate other toxic metals. Cen et al. presented accumulation of different metal, for example cadmium (Cd) in cap and stipe of CC. Amount of Cd in CC increased with the increasing metal level in soil. After CC cultivation on soil with Cd concentration of 0.5 mg/kg, metal accumulation in stipe was 0.27 mg/kg d.w. and in cap was 0.35 mg/kg d.w. (Cen, Hu, & Xu, 2012).

Nickel (Ni) is considered as the one of the largest heavy pollutants (Tang et al., 2016). Accumulation of Ni in the food chain could cause health problem in a human body, such as eczema, respiratory problems, and allergic contact dermatitis (Yeganeh et al., 2013). Tang et al. tested Ni concentration in CC depending on bacterial inoculation, and it was from 5.22 to 15.90 mg/kg. Bioconcentration factor which is content of Ni in CC/Ni concentration in soil was 0.07–0.21 and depended on bacterial inoculation (Tang et al., 2018). Kalač indicated that CC harvested on unpolluted areas may also accumulate aluminum, barium, and vanadium (Kalač, 2016).

There is only little information about toxicity dose of CC in published papers. Ren et al. determined lethal dose LD_50_ (dose which killed half of the subjects) by injection of triglycerides extracted from fermented CC to mice. LD_50_ was estimated as 400 mg/kg b.w. (Ren et al., 2012). Different assay on mice showed that oral administration of alkalic‐extractable polysaccharides from CC in 1,000–6,000 mg/kg b.w. dose was without any toxicity symptoms ( Zhao et al., 2019). Ma et al. evaluated toxicity effect of vanadium (IV and V) absorbed by CC and fermented mushroom of CC on mice. The study consisted of measured body weight and masses organs such as the liver, kidney, and heart. In this test, vanadium (IV and V) absorbed by CC reduced body weight (28.6 ± 2.1 and 20.2 ± 2.6 g), liver weight (1.36 ± 0.06 and 1.23 ± 0.07 g), kidney weight (0.41 ± 0.03 and 0.32 ± 0.05 g), and heart weight (0.16 ± 0.02 and 0.10 ± 0.01 g) compared with control value of body weight (36.9 ± 1.9 g), liver weight (1.40 ± 0.07 g), kidney weight (0.47 ± 0.03 g), and heart weight (0.18 ± 0.01 g). On the contrary, fermented mushroom of CC did not decrease significantly body weight (36.0 ± 1.7 g), liver weight (1.38 ± 0.05 g), kidney weight (0.44 ± 0.02 g), and heart weight (0.18 ± 0.02 g; Ma & Fu, 2009).

## 5 CONCLUSIONS

In this review, for the first time, all information about composition, bioactivity, as well as potential hazards related to the consumption of CC has been collected in order to get to know this species thoroughly. In‐depth study is required to investigate bioactive compounds of CC and its influence on health as well as to ensure its acceptance among consumers. In this connection, it is also relevant to confirm health benefits and safety of CC. In addition to its basic nutritional value, CC could provide pro‐health benefits, which is the characteristic of functional food. Given these points, CC should be considered for use as nutraceuticals, functional foods, and raw materials for medical preparations.

## CONFLICTS OF INTEREST

The authors declare no potential conflict of interest.
